# Aligning the planning, development, and implementation of complex interventions to local contexts with an equity focus: application of the PRISM/RE-AIM Framework

**DOI:** 10.1186/s12939-024-02130-6

**Published:** 2024-02-27

**Authors:** Monica Pérez Jolles, Meredith P. Fort, Russell E. Glasgow

**Affiliations:** 1 Adult and Child Center for Health Outcomes Research and Delivery Science (ACCORDS), University of Colorado School of Medicine, Anschutz Medical Campus, Mailstop F443, 1890 North Revere Court, Aurora, CO 80045 USA; 2 Department of Health Systems, Management and Policy and Centers for American Indian and Alaska Native Health, Colorado School of Public Health, Anschutz Medical Campus, Aurora, CO USA; 3 Department of General Pediatrics, University of Colorado School of Medicine, Anschutz Medical Campus, Aurora, CO USA; 4 Department of Family Medicine, University of Colorado School of Medicine, Anschutz Medical Campus, Aurora, CO USA

**Keywords:** Health equity, PRISM, RE-AIM, Implementation, Complex, Functions and forms, Co-creation, Context alignment

## Abstract

For the fields of implementation science and health equity, understanding and being responsive to local contexts is of utmost importance to better inform the development, implementation, and evaluation of healthcare and public health interventions to increase their uptake and sustainment. Contexts are multi-level and include political, historical, economic, and social factors that influence health, as well as organizational characteristics, reflecting the richness of members’ views, resources, values, and needs. Poor alignment between solutions and those contextual characteristics could have an impact on inequities. The PRISM (Practical Robust Implementation and Sustainability Model) is a context-based implementation science framework that incorporates RE-AIM outcomes (Reach, Effectiveness, Adoption, Implementation, Maintenance) and offers guidance to researchers, practitioners, and their patient and community partners on how to conceptualize, assess, and address contextual domains with a focus on health equity. Drawing from systems thinking, participatory engagement, and health equity principles, this commentary expands on previous work to 1) offer a novel perspective on how to align an intervention’s core functions and forms with the PRISM’s contextual domains, and 2) foster an ongoing and iterative engagement process with diverse partners throughout the research and practice process using a co-creation approach. We recommend intervention-to-context alignment through iterative cycles. To that end, we present the RE-AIM Framework’s ‘outcomes cascade’ to illustrate touch points of opportunity and gaps within and across each of the five RE-AIM outcomes to illustrate ‘where things go wrong’. We present a case study to illustrate and offer recommendations for research and practice efforts to increase contextual responsiveness, and enhance alignment with context before, during, and after implementation efforts and to ensure equity is being addressed. We strive to make a conceptual contribution to advance the field of pragmatic research and implementation of evidence-based practices through the application of the contextually-based PRISM framework with a focus on health equity.

## Background

There is increased priority by communities, researchers, practitioners and funding agencies to design, implement, and evaluate interventions from a health equity and participatory focus [1, 2]. Having a good understanding of the local contexts where interventions are implemented is crucial to meeting this health equity goal [3]. Local contexts reflect not only the physical setting, richness of values, needs, priorities, resources, and healthcare systems, but also the political, historical, economic, and social factors that influence health [4].

We define intervention broadly. Our definition includes any program, training, treatment, policy, action or implementation strategy (i.e., activities supporting the adoption, uptake and sustainment of the intervention) [5] taken to prevent or treat disease, or improve health in other ways [6]. We also focus on a systems perspective to understand how complex interventions align with local dynamic contexts, and are centered on equity and inclusion. Complex interventions refer to those with multiple and interacting components that require coordinated action among implementers and flexibility in their implementation to capture the dynamic nature of diverse local contexts [7–9].

A co-creation engagement with diverse partners such as patients, caregivers, community members, policymakers and professional practitioners is needed to fully understand key contextual characteristics [10, 11]. For example, clinical personnel (e.g., providers, managers) can offer feedback on how an evidence-based intervention is best delivered within a local context and based on its characteristics (e.g., resources, infrastructure), as well as patients on how the intervention is presented and received. This contextual understanding is critical because even if an evidence-based intervention is available and shown to be effective, its impact could be limited by an unsuccessful and inequitable design, implementation, and evaluation. A contextual understanding can also foster an equitable implementation process and ultimately contribute to the attainment of equity outcomes (e.g., Reach and Representativeness) that matter to implementers (e.g., clinicians, public health staff) as well as to patients, caregivers, and communities impacted while preventing or reducing unintended consequences.

Implementation Science (IS) can offer guidance on aligning interventions to local contexts with an equity focus. IS is the study of “…methods to promote the systematic uptake of research findings and other evidence-based practice into routine practice, and hence, to improve the quality and effectiveness of health services.” [12]. IS underscores a need to understand local contextual domains such as community members’ priorities, needs, and values as well as organizational perspectives, resources, and infrastructure because these factors impact an intervention’s uptake, equitable implementation, and impact [13–15]. The IS emphasis on context is reflected in the use of frameworks and models to guide the identification of these key contextual domains [16].

The concept of engaging clinical and community partners in research and implementation efforts is not new. There are important contributions from adjacent fields such as health equity research [17, 18], CBPR [11, 19], and from methodology within the implementation field such as mapping [20, 21] and participatory systems modeling [22, 23]. Our approach broadens and contributes to these efforts by combining a pragmatic IS framework (PRISM) with a systems science-based concept (function-form), and emphasizing iterative application of a monitoring process for intervention-context alignment through a ‘RE-AIM cascade’ described below. Collective reflection on these iterative cycles may be aligned with Oscar Jara’s concept of the ‘Systematization of Experiences’ which applies critical reflection to better understand experiences within their historical context and link research and action in a single participatory process [24].

This paper proposes a model of interactions that could enhance an equitable implementation process. We expand our previous work from co-authors and others [4, 25–27] on the application of the Practical, Robust Implementation and Sustainability (PRISM) model from equity and systems perspectives. Our goal is to offer guidance to researchers and practitioners on the use of a) PRISM to align contextual needs with the design and evaluation of healthcare and public health interventions from a systems thinking perspective, b) a co-creation engagement approach to actively inform that alignment process [28], and c) the RE-AIM ‘Outcomes Cascade’, framed as a way to inform and support a formative evaluation, to achieve an intervention-to-context alignment using reflective micro-cycles. To illustrate these approaches, we present two case examples, discuss lessons learned, and provide suggestions for future research and practice.

### The Practical Implementation Sustainability Model (PRISM)

**Fig. 1:**
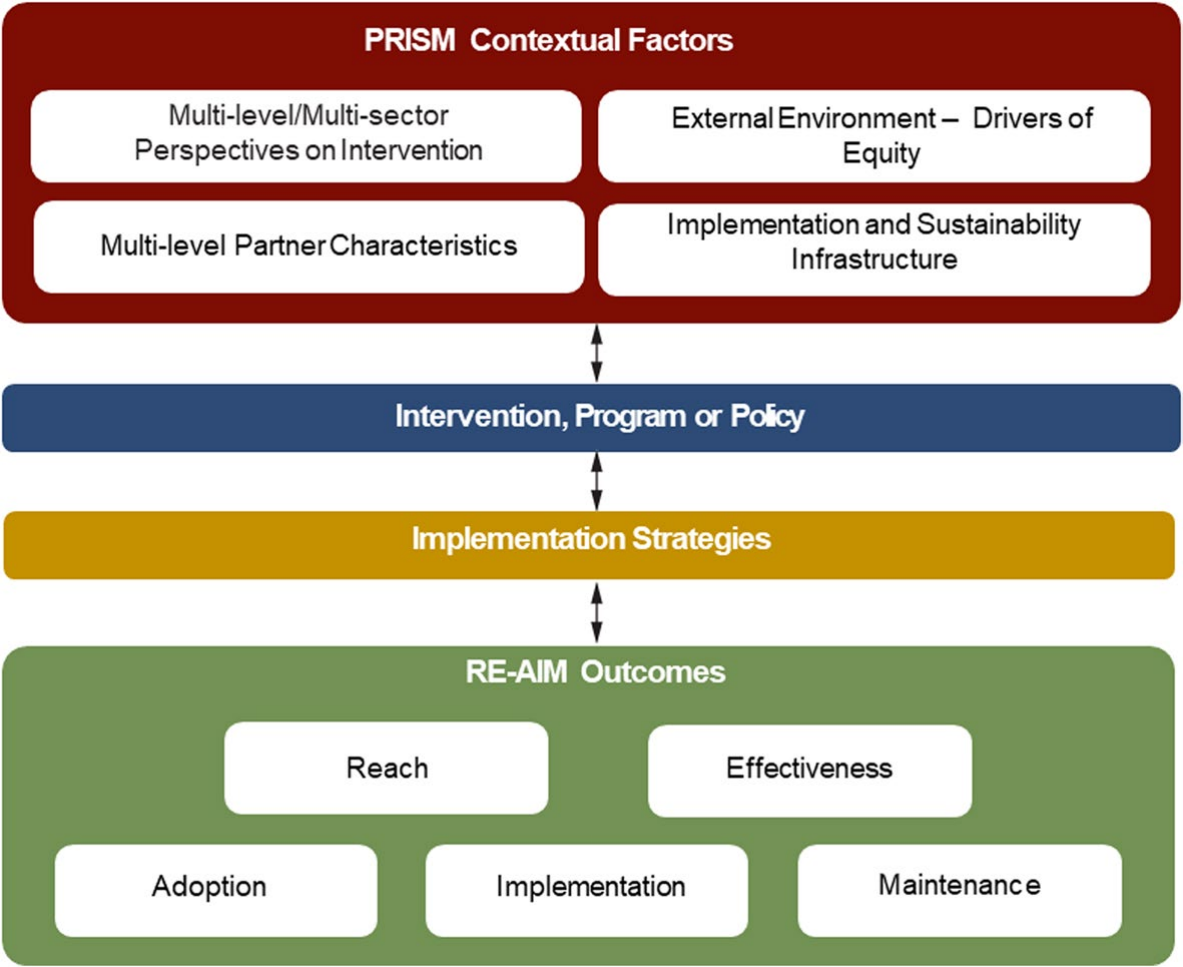
Illustration of the Practical Implementation Sustainability Model (PRISM) and RE-AIM Outcomes

PRISM was developed as a pragmatic and intuitive model to improve translation of research-tested interventions into health systems practice and ultimately population health impact [29]. PRISM is a context-oriented IS framework that can guide researchers and practitioners to understand, assess and address structural drivers of health inequities and be better informed to address them during design, implementation, and evaluation. PRISM is both a determinant and evaluation framework in the classification suggested by Nilsen [30] and has more recently also been used as a process framework in that is used in planning, implementing and evaluating projects as discussed in detail in Glasgow et al. (2019) [31] and Holtrop et al. (2021) [32]. As the top of Fig. 1 illustrates, PRISM addresses context by considering how 1) *perspectives* on the program, policy, or intervention; 2) the external environment; 3) the implementation and sustainability infrastructure; and 4) the *characteristics* of those involved in delivering and receiving a program influence program adoption, implementation, and maintenance.

Within the intervention domain, PRISM incorporates the *perspectives* of community members who receive the intervention, and the implementation staff members – or implementers of an intervention—at different levels of influence (e.g., those in leadership positions, mid-level managers, and frontline staff who interface more directly with the public) [33] to help understand what factors need to be considered and addressed for successful implementation and sustainment of complex interventions [34, 35]. Inclusion of the Implementation and Sustainability Infrastructure domain was based on experience in healthcare settings in which those settings that were able to implement and sustain programs most consistently had infrastructure and support processes and resources to deliver the intervention.

As shown in Fig. 1, the PRISM contextual domains along with the selected implementation strategies are conceptualized to impact five RE-AIM implementation outcomes in the bottom of the Figure [32, 36, 37]. Space limitations preclude detailed review of the more widely known RE-AIM outcome dimensions However, we highlight two issues that are especially important for equity. The first is that PRISM includes RE-AIM- it is an extension of the earlier RE-AIM framework that adds contextual domains hypothesized to impact RE-AIM outcomes- rather than a different framework. The second point concerns representativeness (or equity) of results on the various RE-AIM outcomes. It is important to stress that representativeness- or equity- is important across all RE-AM dimensions- not just reach as is most commonly reported [38, 39].

The PRISM contextual domains pragmatically identify key factors relevant to equity. and when addressed, can enhanced equity within broader socio-political-historical-structural contexts that sustain or challenge inequities [4]. We also encourage ongoing assessment of PRISM domains during the pre-implementation, implementation, and post-implementation phases of specific factors relevant to equity. Using PRISM allows for equity and sustainability planning from the outset by acknowledging a need for tailoring implementation efforts and resources in a way that maintains equality and fairness [40].

**Fig. 2:**
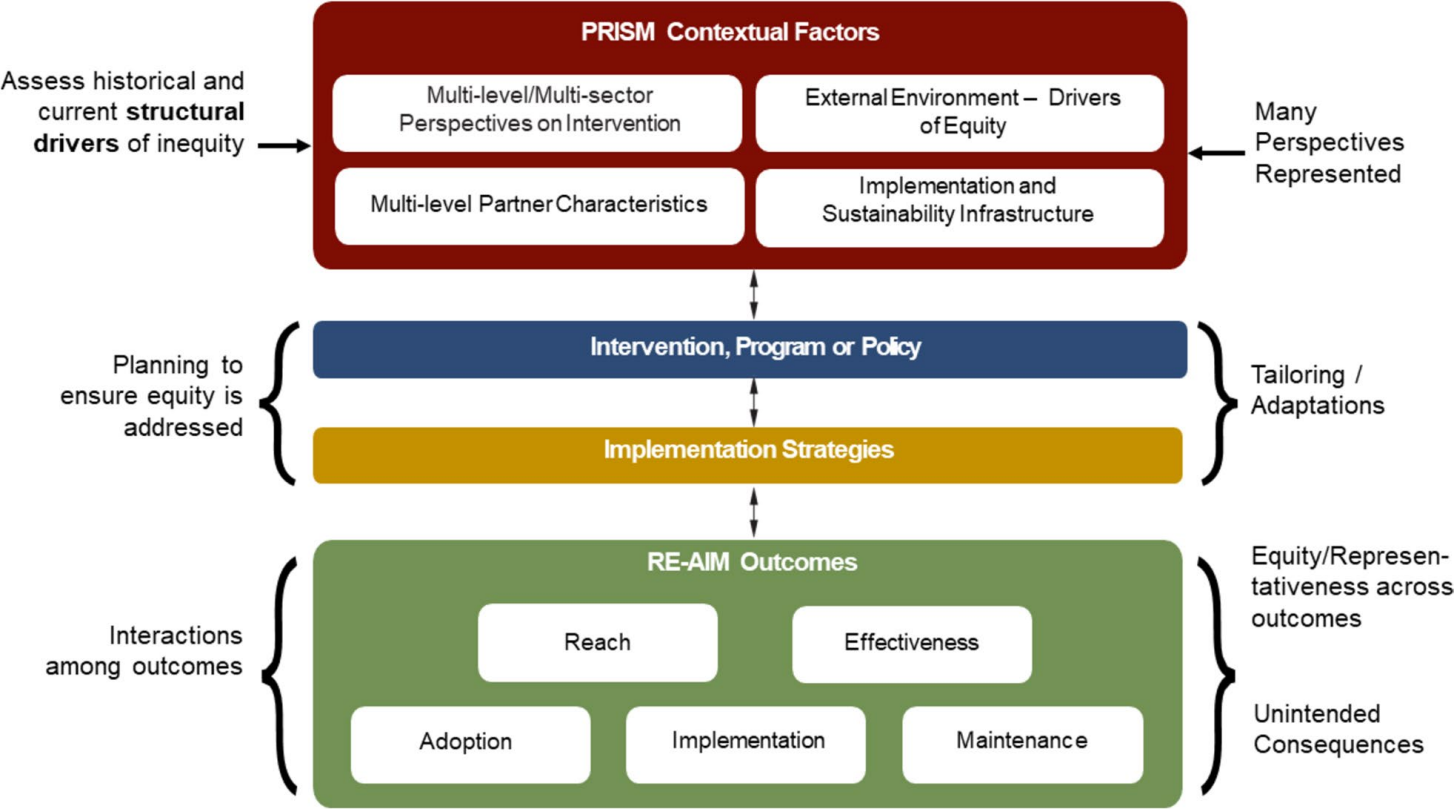
PRISM (and RE-AIM Outcomes) with an equity lens

As RE-AIM outcomes are inter-dependent, an intervention’s impact in one outcome may trigger cascading changes in other outcomes (e.g., uneven Adoption of interventions leading to unintended consequence of selective Reach). Thus, adaptations and tailoring are almost always needed through iterative cycles to address interdependence, and avoid unintended consequences [41, 42]. Tailoring can include local changes in policy, or a need to address potential unintended consequences early on as a way to increase the cultural and equity relevance of the intervention. See Fig. 2 depicting previous work on applying the PRISM and RE-AIM model to include equity considerations and that we use as a starting point in this paper.

### Use of PRISM to align interventions to local contexts from a systems perspective

Most often, complex health-related interventions are implemented in dynamic and diverse contexts [7, 43, 44]. Thus, the ongoing process of fit or alignment to local dynamic contexts is critical to the successful design, adoption, implementation and sustainment of health-related interventions [45]. Yet, there is a lack of guidance on specifying exactly *how to align interventions to local contexts.* We propose the use of two concepts from the complex systems literature to align intervention’s core functions and forms to contextual characteristics. An intervention’s *core functions* refer to “what the intervention was intended to do” [46], the structural or procedural goals designed to meet system and/or patients’ needs [7]. Using PRISM to align an intervention’s core functions to local contexts is critical to preserving the integrity and fidelity of the intervention and the change process [7, 46]. For example, for a clinical intervention where families are screened in primary care for psychosocial needs, a core function of that intervention is of increasing caregiver awareness and knowledge of the impact of toxic stress and trauma on children’s development. This function or goal could be negatively impacted by a history in the United States of negative experiences between minoritized families and local enforcing child protective service agencies [47].

An intervention’s *forms* refer to a flexible menu of activities, procedures, steps or implementation strategies that local communities or healthcare systems adopt to carry out a core function, to make it work for them in their setting (e.g., webinars, informational handouts, peer coaching, website). Forms can also be considered to be context-dependent adaptations or implementation strategies. They are also dependent on the level of function from a particular intervention (e.g., broad/macro-level public health function versus a specific health prevention education function). Forms then can change to adapt to the dynamic and diverse nature of local contexts, while preserving fidelity to the intervention’s core functions [7, 46]. Thus, Functions and forms are “ways to conceptualize an intervention’s change process (or mechanism) and specify its components…consistent with theory.” (P. Hawe, personal communication, October 11, 2023). In the example provided, forms could include trained peer partners during the implementation of pediatric screenings and subsequent service referrals to support families and overcome mistrust. Pérez Jolles and colleagues have suggested the creation of an aligned need-function-form matrix as a tool to guide this alignment process [7].

### Co-creation engagement approach: Better informing the intervention-to-context alignment process

A key to a successful implementation of interventions is to tailor their implementation to local contextual characteristics. Thus, an active and participatory engagement with diverse partners that include patients and community members is suitable to better inform that tailoring. More specifically, researchers’ and practitioners’ efforts to promote a participatory co-creation engagement [48–50] approach throughout a research/practice process can ensure that the planning, development, and implementation of interventions reflect the needs, resources, priorities and values of local communities and healthcare systems [28, 51]. Co-creation with a focus on equity has been used in other fields (e.g., equity research, anthropology, civic engagement, community psychology and prevention science) [52–55] and defined asa group process where all partners actively share their knowledge, experiences, skills and resources [28, 51, 56]. It is important for the field of Implementation Science to learn from these fields with a history of using this concept of co-creation in the context of anti-colonial scholarship [57, 58].

We conceptualize co-creation as an overarching type of engagement, within the spectrum of Community-Based Participatory Research (CBPR), where researchers create the group conditions for all partners to contribute to building the knowledge and direction of all or some of these areas: (a) needs assessment and priority selection, (b) the intervention (e.g., design and development), (c) the research study or evaluation process, and (d) the implementation and dissemination plan [59–61].

Co-creation is well suited to inform the alignment of intervention-to-context process because historically under-represented and minoritized partners are empowered to share their experiences, preferences, and needs, be part of priority selection, and actively contribute to the knowledge built and to inform the direction of the research or practice process [28]. Previous research have identify five core functions of co-creation engagement that are rooted in equity, inclusion and justice (i.e., equity in relationship building, spaces for reflexivity, reciprocity and mutuality, personalized and transformative engagement experience for partners, and diverse and open social networks) [28]. From this perspective, a co-creation engagement is a multi-level engagement effort implemented in dynamic contexts. As such, the concepts of core functions and form is compatible with a co-creation engagement. Co-creation core functions (principles or goals) and concrete forms have been identified from the literature and discussions with national researchers and community members [50] as shown in Table 1 [28].

**Table 1:**
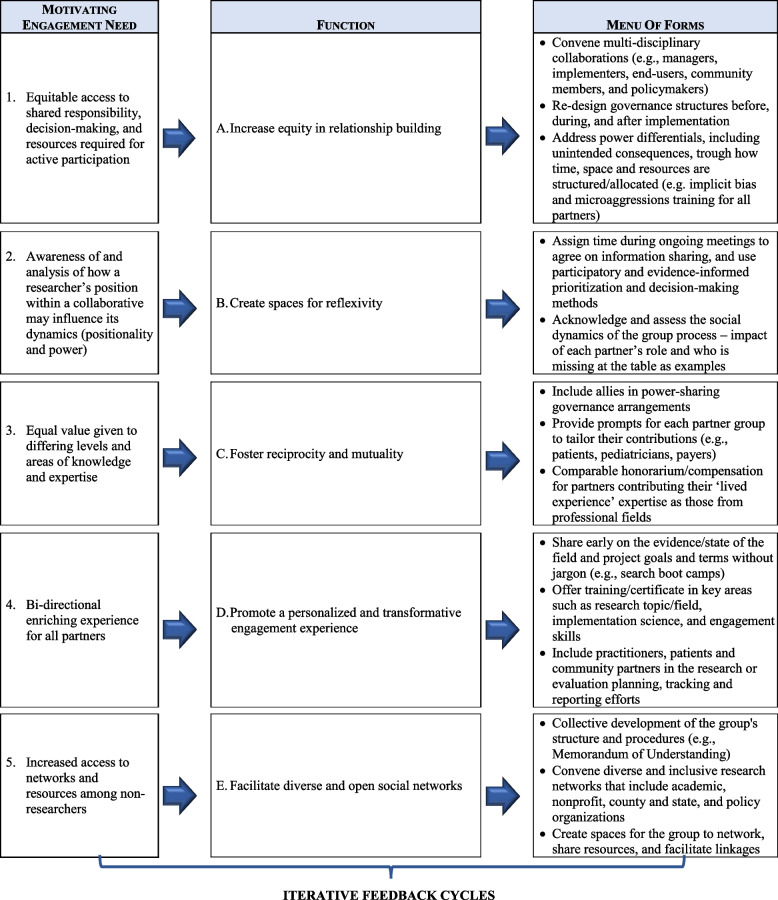
Functions and forms matrix for a co-creation engagement strategy in engaged research

It is essential to structure a co-creation engagement through clear and iterative steps. We have implemented the following steps in our work [62]: (a) careful planning of the group process to promote co-creation core functions through concrete forms (Table 1); (b) perform a needs assessment with partners and informed by a framework such as the PRISM to align intervention to context; (c) design tailored strategies to support the implementation of the intervention; (d) evaluate the group process and its impact on implementation and outcomes. In addition, we acknowledge that engagement is an active process of activities among partners, and that it is important to consider the PRISM contextual domains continuously during this process to actively reflect on context at all times and to understand how context influences the implementation experience.

**Fig. 3:**
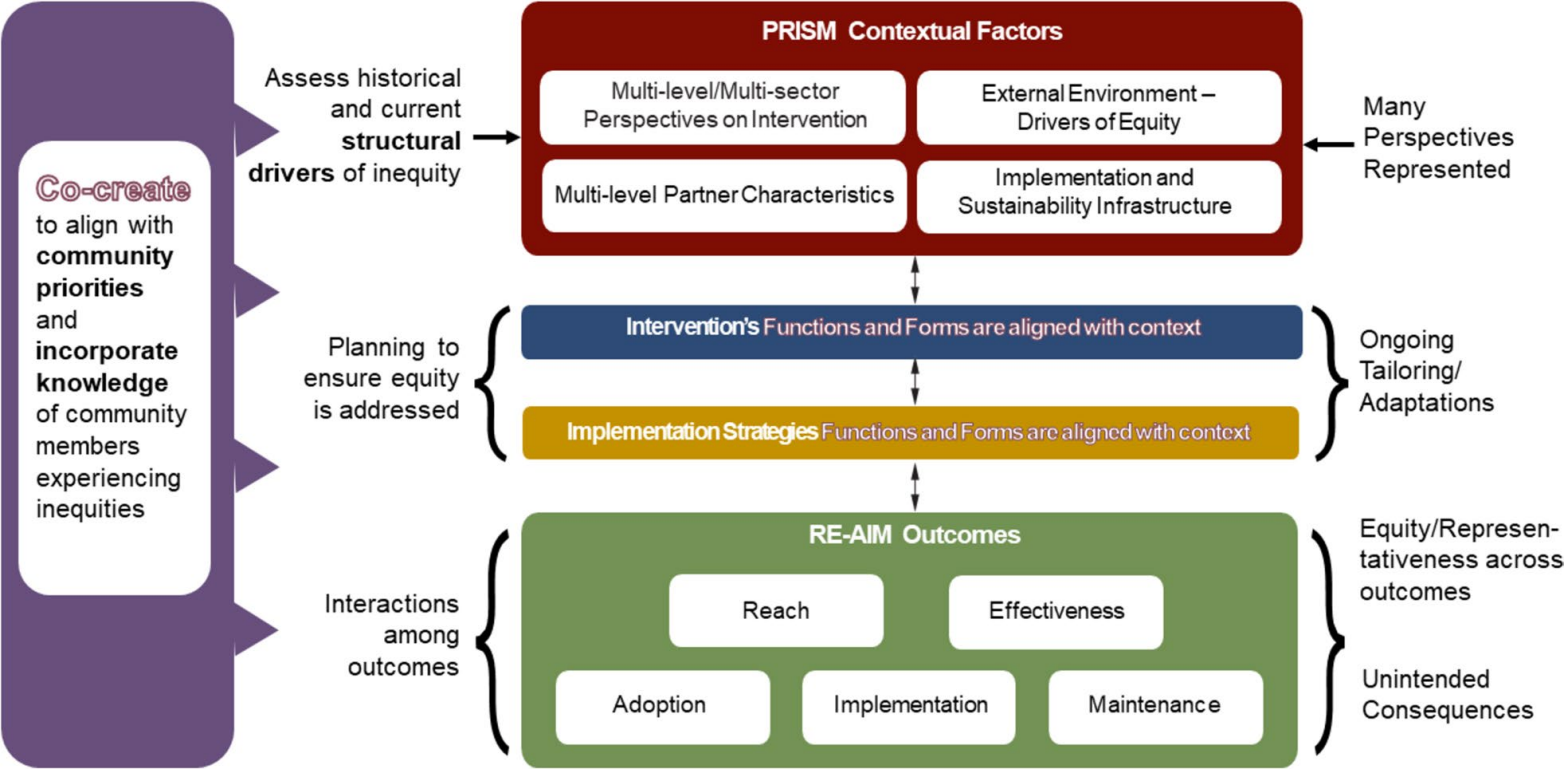
PRISM (and RE-AIM Outcomes) from a systems and co-creation perspectives

PRISM explicitly encourages strong representation of these groups throughout the research or practice process [38]. Formalized governance structures and accountability mechanisms such as memoranda of understanding and transparent budgeting can help address power differences among groups such as academic researchers and community partners. Last, a co-creation engagement can also be used to identify and measure (RE-AIM) outcomes in a way that matters to patients, families, and other partners. A co-creation engagement that includes the voices and priorities of patients and community members is not confined to a single implementation phase or area [38]. This participatory engagement should start early on and inform every aspect of the planning, design, implementation and evaluation of interventions (Fig. 3).

### Context-intervention alignment using ongoing cycles: the RE-AIM Outcomes Cascade

**Fig. 4:**
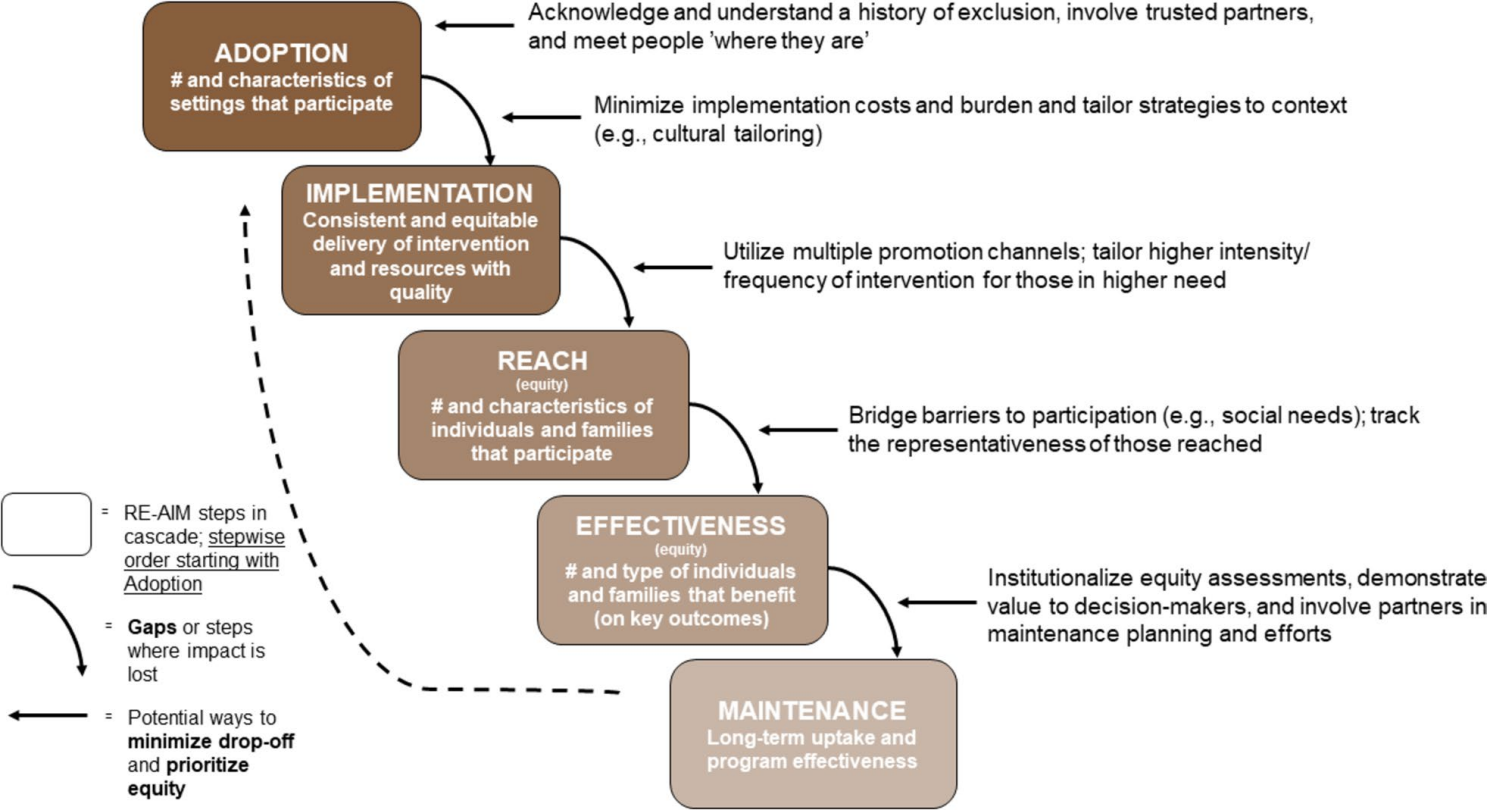
RE-AIM Outcomes Cascade starting with adoption

The implementation of interventions in dynamic contexts requires an ongoing monitoring of the intervention-to-context alignment [7, 43]. This process is essential in promoting translational research because, when absent, an intervention can start with high potential for impact, but may gradually lose this potential at multiple key steps throughout the research process due to loss of clinical sites, study participations, implementation challenges or failure to sustain. We recommend managing this monitoring process through iterative implementation action cycles. To that end, we present the *RE-AIM Outcomes Cascade* in Fig. 4 to guide such cycles and with a focus on contributing to equitable and sustainable outcomes.

This figure illustrates the importance of considering sequential and multi-level impact at various stages of program planning and implementation. There are a number of ‘steps’ involved in a successful and equitable intervention. This begins with Adoption (the A in RE-AIM) in terms of which settings will elect to try an intervention. This is followed by Implementation planning and initiation of the delivery process. The next ‘step’ that is involved is the extent to which implementation (specifically recruitment) activities result in equitable reach among different subgroups. We acknowledge the sequence-iterative nature of the cascade and that is not a strictly unidirectional process (e.g., arrow from the RE-AIM dimension in the figure back to first) but recognize that it is helpful to understand the “voltage drops” that may occur with response to equity in an implementation experience. That is, there is a general sequence of steps (e.g., if Reach is low or there is no adoption of an evidence-based intervention, it cannot be effective or sustained) while also stressing the need to foster learning cycles (e.g., what it is learned from going through each of the steps informs new cycles of learning how to best adopt a new intervention and so on).

Importantly, at each of these ‘steps’, there is a potential for drop off or loss of impact across settings and individuals if key equity issues are not addressed at each step. For example, during the COVID-19 pandemic, the implementation of telehealth increased dramatically as a way to overcome social distancing public health policies during the pandemic. Yet, a history of inequities on access and trust on technology among patients [63, 64], as well as implementation efforts not ‘meeting some patients where they are’ created inequities on the Reach of this intervention. Evidence showed that Latinx and Asian populations were less likely to use telemedicine compared to other racial and ethnic groups [65]. This unequitable Reach in turn impacts the number and type of individuals benefiting from Telehealth during the pandemic. Thus the ‘cascade’ effect. The good news is that at each step there are also actions that can be taken to prevent or minimize drop-off or inequities at each step as noted in Fig. 4.Altough each RE-AIM outcome is important, they need to be integrated to estimate cumulative health equity impact. We recommend periodic assessments of progress on prioritized RE-AIM outcomes using brief pragmatic items completed by key team members to inform necessary adaptations [42, 66].

As programs cycle through these steps (on an annual basis for ongoing/sustained programs, for example), the RE-AIM cascade may continually enhance or reduce their focus on equity in future iterations. This may be thought of as positively reinforcing equity (a virtuous cycle) or a negatively reinforcing cycle that limits equity. The cascade underscores the importance of ongoing contextual insight and the need (and opportunities) to plan for infrastructure improvement, resource distribution, and policy changes to address persistent or emerging gaps and societal inequities at the various ‘steps’.

Throughout these ongoing cycles, information is fed back to all partners (e.g., committees, advisory boards, research or evaluation teams, patient and advocacy groups) so they can learn from the engagement process, capture contextual changes as they happen and adapt with the goal of informing future implementation efforts. These multiple learning cycles offer opportunities for a critical reflection process to gain perspective and understand experiences within the broader historical-social-political-cultural process/structures (or “the system”) [67].

**Fig. 5:**
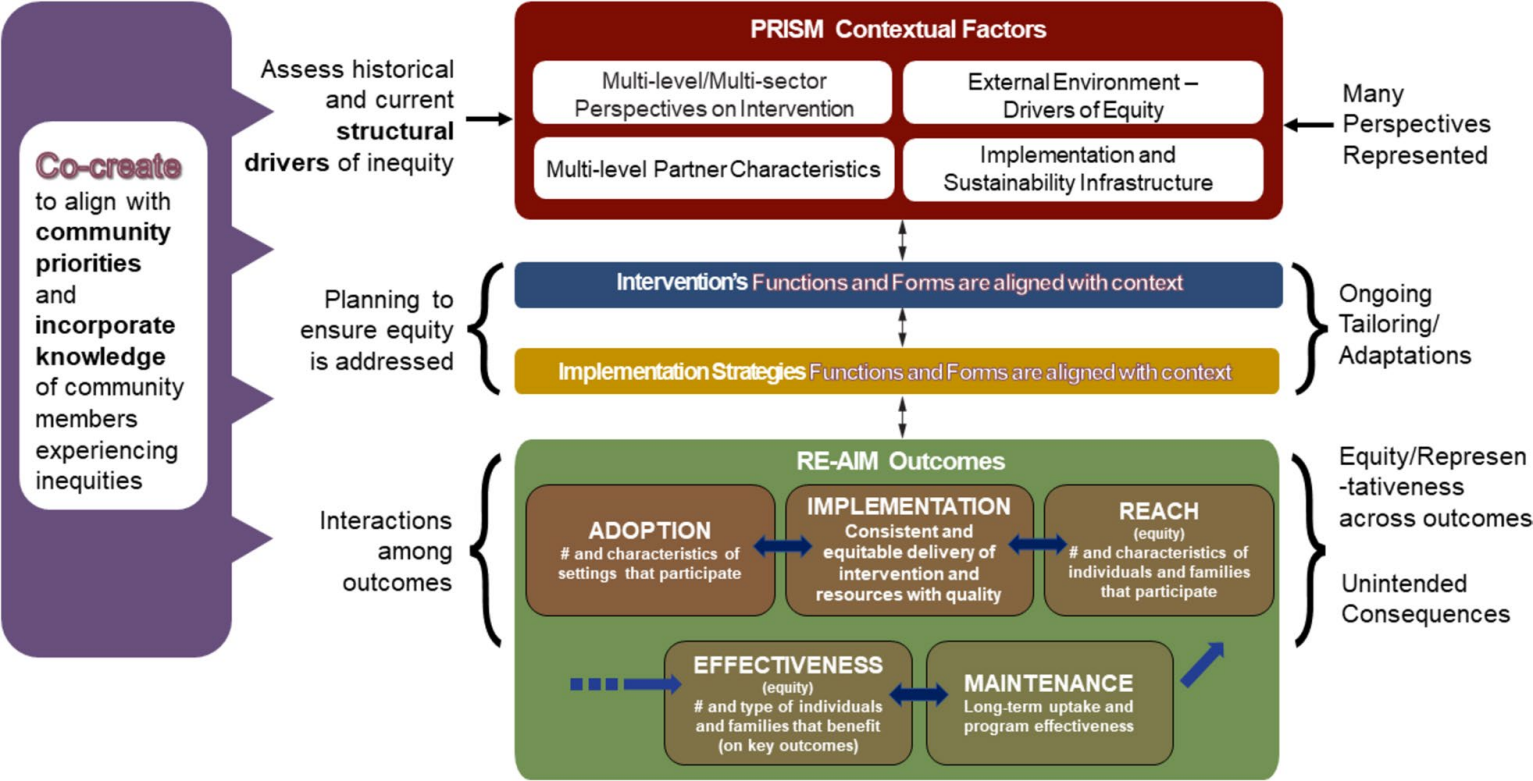
Integrated PRISM (and RE-AIM Outcomes) from a systems and equity perspective

Researchers and practitioners can assist change efforts by documenting context prior to, during, and post-implementation – in each cycle of the RE-AIM cascade [45]. Ideally, an equity lens should simultaneously consider: 1) the cycle of implementation and 2) the context, recognizing that efforts to promote equity on both will be mutually reinforcing. See Fig. 5. It is important to note that although the arrows in the RE-AIM outcomes cascade are unidirectional in this figure, but we conceptualize it as an ongoing cycle that does not end with maintenance but a process that inform and feed back into the next iteration – and into our broader learning that we bring to future implementation efforts.

### Application of concepts: illustration with a case example

We illustrate the various areas discussed in this paper through a case example as described in this section. See Table 2 for a summary of these applications.

**Table 2:** Intervention-to-context alignment processes using an example emphasizing key areas of illustration

	**Engaging patients and clinicians to co-create feasible and sustainable approaches to implement evidence-based cancer control**
***PRISM-relevant contextual domains***	• **Organizational perspective:** Inclusion of oncology service line personnel at multiple levels to inform the design, planning, and implementation of the IA3-CP intervention • **Patient perspectives:** Inclusion of patients with an existing cancer diagnosis during the planning and design of the intervention • **External environment:** The availability and type of local community resources (e.g., transportation) to support patients’ needs, preferences, and lifestyle changes inform the ‘linkage’ aspect of the tool intervention design
***Use of co-creation, informed by functions and forms concepts***	• A **Steering Committee** comprised of clinic personnel was convened starting in the grant proposal writing phase to inform the feasibility of the intervention and key areas to consider in the research study design and planning phase • Clinic and patient partners participated in **co-creation workshops**. The intervention’s core functions were presented as a starting point and partners worked on refining those forms and on tailored forms (e.g., clinic workflows)
***Equity and use of the RE-AIM Cascade***	• Inclusion of **diverse patient partners in co-creation workshops** to empower their decision-making early on, inform adaptations, and include multiple perspectives • Feedback loops formally established across workshops to address potential drop-offs at different steps and prioritize equity (e.g., patients with new a cancer diagnosis and experiencing high levels of distress and low social support not using the tool)

### Example: engaging patients and clinicians to co-create feasible and sustainable approaches to implement evidence-based cancer control

To guide cancer treatment decisions for older adults, geriatric and patient-centered risk assessments are recommended — including social determinants of health (SDoH) and behavioral risk factors that influence patient care. Such assessments can guide treatment discussions, inform the intensity of treatment, and identify supportive care needs; yet are not implemented routinely in oncologic clinics. The following pilot study example is guided by PRISM and uses co-creation engagementwith a multi-perspective steering committee, clinic-based workshops, and a diverse group of patients to inform and adapt a patient-centered web based multiple risk assessment and feedback system [68]. The goal is to use this diverse feedback during the planning and design of a screening tool to integrate screenings in areas relevant to older adult cancer patients in a way that is feasible, actionable, and sustainable. The resulting package is termed the “Integrated Aging Assessment for Action in Cancer Patients” tool or IA3-CP.

#### Use of PRISM contextual areas to inform the design of IA3-CP

The intervention’s design and implementation plan have been informed by PRISM contextual areas. For example, during the planning phase of the project (6 months), the organizational resources, capacity and perspectives from oncology clinics, through multiple partner roles such as providers, frontline support staff, and managers were included through 60-min co-creation workshops. During these sessions, we learned that the clinics preferred to implement the IA3-CP tool online of the tool online first to build a tailored workflow and capacity, and then add in-person implementation at the clinics. Partners actively co-created the IA3-CP intervention’s design and tailored clinic workflows to successfully embed the new intervention into the daily clinic routine [61].

#### Use of co-creation, informed by functions and forms concepts, to engage partners

The co-creation process began by convening diverse partners over several months during the pre-pilot funding phase. A steering committee included members of oncology clinics with representation from multiple roles (providers, managers, support personnel, and health system programmatic leadership). This group provided guidance to the research team on IA3-CP core functions (goals) and forms (actions) to inform the design and planning of the tool. The research team used that feedback to convene 60 to 90-min workshops with clinic personnel and patients. Sessions were held remotely using Zoom and facilitated by a clinician champion and by the first author. During the sessions, all partners co-created or refined IA3-CP core functions (goals) and forms (e.g., workflows with procedures to introduce the tool to clinics) with the goal of offering patients an equitable, coordinated, and patient-centered experience from screenings to referrals.

#### Focus on equity and use of the RE-AIM Cascade

The research team recruited a group of diverse patient partners (*n*= 7) based on ethnicity, language, race, cancer diagnosis, and place of residence. We included them early in the research process because patients, including those from minority backgrounds (i.e., race, ethnicity and older adults), have been historically excluded from research efforts, including clinical trials, and intervention planning and design efforts [69].

The patient partners for this study had no previous participation in clinical research or advisory boards. They were included in the crucial planning phase when key decisions were made, and to make sure multiple perspectives were represented. Partners participated in co-creation workshops lasting 45–90 min each in English and Spanish. Sessions were co-led by the first author and a clinician champion, as a way to actively inform how the intervention is presented to patients and to identify strategies to increase patient comfort and trust. Sessions focused on co-creating with patients the content included in the tool modules, language, and ways to make the tool user friendly. Examples of how partners co-created the IA3-CP tool include patients sharing with researchers that a cancer diagnosis can be traumatic for many patients and the recommendation for inclusion of a mental health section in the tool. Researchers fully adopted this recommendation and worked with the tool designer to add this information in a new module of the IA3-CP online tool intervention to acknowledge the distress and potential trauma that a new cancer diagnosis may have on a patient. In addition, based on patient feedback, we designed the system to be more flexible so patients could save their responses and complete the tool at their own pace. Summaries of each session were created by the project manager, based on detailed notes from each meeting with patients, and then shared with researchers for discussion and action. This action in turn was shared back with patients at the next co-creation session and using one or two iterative cycles.

## Discussion

We propose an integration of PRISM, systems thinking perspective, and a co-creation engagement approach to guide practitioners and researchers on the alignment of complex interventions to local contexts that is centered on inclusion and equity. It is critical that researchers and practitioners complement integration efforts with pragmatic assessments that allow partners to evaluate the extent to which engagement has been equitable and co-creation has occurred [70]. Researchers and community partners can often have very different perceptions about how much this has happened.

This work contributes to the field by offering conceptual guidance on the application of the PRISM model to align intervention to context and with an equity focus through the use of complex systems concepts and a co-creation engagement with partners. PRISM is a suitable model to address equity because it is context-based and offers a practical and straightforward way for researchers and practitioners to address four contextual domains that have been shown to impact equity (e.g., structural domain with a history of lack of access to quality education, healthcare and economic opportunities for women, and among racial and ethnic minority groups in the United States) [71, 72]. This first step of elucidating conceptual linkages and practical application of the PRISM model can guide and inform future empirical efforts to test them. Although we have focused on the integration of co-creation with the PRISM framework, similar integration could likely be achieved with other implementation science frameworks.

This paper offers novel recommendations to this alignment process. First, a co-creation research engagement that supports the alignment of contextual needs, values, and resources to an intervention’s core functions and forms can better inform participatory research including which dimensions of equity will be prioritized (e.g., representativeness of participants reached by the intervention; or incorporation of equity-focused measures into the tracking/evaluation system) [73]. Second, we emphasize representativeness and reporting across all five RE-AIM outcomes and not simply reporting the most often used outcome (e.g., Reach (in its most basic form—the number of participants) [74]. Third, the concept of the RE-AIM ‘Cascade’ and examples of actions that can be taken to prevent or mitigate inequities that can emerge at any step in this sequential implementation cascade is novel. Finally, the emphasis on iterative applications of PRISM as operationalized most recently in the iterative PRISM (iPRISM) webtool [73] noted above is essential for long term success and sustainment of equitable outcomes [75].

### Strengths and limitations

Strengths of this paper are based on the integration and expansion of previous work in IS and systems science. Specifically, it illustrates application of PRISM, function-form, and co-creation models and approaches to address health equity. As illustrated in the figures, related functions and forms publications and iterative RE-AIM resources are relatively accessible, concrete and understandable to community representatives. The concepts, approaches, and recommendations we summarize have not been compared to alternative approaches. Further work is needed to address issues such as 1) a need to further test the proposed relationships to enhance equity, including engagement (equitable partnership) and considering equity in all phases of the implementation process; 2) the optimal composition of multi-level community partner groups for different projects 3) the costs of implementing these methods; and 4) the types of settings, problems, and partners with which these approaches work best. Finally, the RE-AIM Cascade concept has conceptual appeal, it has not been tested and the optimal number of cycles that are most cost-effective are not known.

### Recommendations for research and practice

We recommend additional research on several key areas discussed. First, research on key PRISM domains and how they relate to equity is needed such as the study of whether some domains are more strongly related to certain aspects of health equity (e.g., the domain of ‘Patient Perspectives’ may be more strongly related to equitable reach than other PRISM domains such as the Implementation and Sustainability Infrastructure). There is a need for more specific guidance for practitioners on the amount of time, facilitation, and guidance needed. More research is needed on implementation costs to apply PRISM co-creation engagement methods. Last, we ponder whether pragmatic measures of co-creation can be developed and validated, and whether greater intensities (dose) of recommended co-creation engagement are associated with more equitable intervention implementation and outcomes.

## Conclusions

IS emphasizes a need to understand local contexts through ongoing participatory engagement to improve service delivery and address disparities. PRISM is a pragmatic accessible IS framework that can guide research efforts seeking to embed local contexts’ characteristics, including multi-level drivers of equity, into the design and evaluation of interventions. This ‘context to intervention alignment’ can help complex health interventions to achieve their goals [34]. PRISM and its iterative ‘RE-AIM Cascade’ component can help programs achieve key implementation outcomes (e.g., reach, adoption, sustainability) with an explicit focus on equity. In summary, we address future directions for the application of the PRISM RE-AIM framework [76] by presenting a concrete way to align an intervention to local contexts using a complex systems perspective with the concepts of functions and forms. We propose the use of co-creation processes in health-related research because it is a type of engagement to inform this alignment process, and contribute to equity being addressed. Therefore, ‘there is no equity without engagement, and no engagement without equity’.

## Data Availability

No datasets were generated or analysed during the current study.

## Abbreviations

IS: Implementation Science
PRISM: Practical Robust Implementation and Sustainability Model
RE-AIM: Reach, Effectiveness, Adoption, Implementation, Maintenance
CBPR: Community-Based Participatory Research
iPRISM: Iterative Practical Robust Implementation and Sustainability Model
IA3-CP: Integrated Aging Assessment for Action in Cancer Patients
